# Beyond allergic progression: From molecules to microbes as barrier modulators in the gut-lung axis functionality

**DOI:** 10.3389/falgy.2023.1093800

**Published:** 2023-01-30

**Authors:** Jorge Parrón-Ballesteros, Rubén García Gordo, Juan Carlos López-Rodríguez, Nieves Olmo, Mayte Villalba, Eva Batanero, Javier Turnay

**Affiliations:** ^1^ Department of Biochemistry and Molecular Biology, Faculty of Chemistry, Complutense University of Madrid, Madrid, Spain; ^2^ The Peter Gorer Department of Immunobiology, King's College London, London, United Kingdom; ^3^ The Francis Crick Institute, London, United Kingdom

**Keywords:** allergy, asthma, epithelial barrier, exposome, food allergy, gut-lung axis, microbiota, SCFAs

## Abstract

The “epithelial barrier hypothesis” states that a barrier dysfunction can result in allergy development due to tolerance breakdown. This barrier alteration may come from the direct contact of epithelial and immune cells with the allergens, and indirectly, through deleterious effects caused by environmental changes triggered by industrialization, pollution, and changes in the lifestyle. Apart from their protective role, epithelial cells can respond to external factors secreting IL-25 IL-33, and TSLP, provoking the activation of ILC2 cells and a Th2-biased response. Several environmental agents that influence epithelial barrier function, such as allergenic proteases, food additives or certain xenobiotics are reviewed in this paper. In addition, dietary factors that influence the allergenic response in a positive or negative way will be also described here. Finally, we discuss how the gut microbiota, its composition, and microbe-derived metabolites, such as short-chain fatty acids, alter not only the gut but also the integrity of distant epithelial barriers, focusing this review on the gut-lung axis.

## Allergy: a pandemic of the 21st century

Allergic diseases are characterized by immunological alterations that drive to hyperresponsiveness mainly against protein components called allergens. These molecules are present in a plethora of natural sources (house dust mites, molds, pollens, bee or wasp venoms, vegetable and animal foods, etc.), and lead to many different inflammatory diseases such as asthma, allergic rhinitis, food allergy (FA) or atopic dermatitis (AD) (1).

Worldwide prevalence of allergic diseases has dramatically increased during the last decades (2–5). Asthma, food allergy, and atopic dermatitis are considered the most relevant allergic diseases, and the connections to each other are still under research. Asthma is a chronic disease where the allergen avoidance is often difficult. Proper diagnosis and follow-up are required, especially in patients displaying severe symptoms, whose main treatment implies the administration of corticosteroids, adjusting doses to the minimum effective ones and/or controlling disease exacerbations (6).

Food allergies affect around 5%–8% of global population, and is increasing every year, causing deaths, alteration of life quality and health cost (7, 8). Nowadays, food allergy development is mainly explained by the “dual allergen exposure hypothesis”, which addresses that food allergen exposure through inflamed skin before exposure to the alimentary tract might lead to the development of FA (9). Accordingly, avoidance of allergenic foods during pregnancy until the first year of the child's life has been widely recommended from decades (10). However, more recent studies suggest that this practice induces an increase on IgE sensitization (11–13). Conversely, the introduction of food allergens during early life could even induce immune tolerance, as concluded by the PreventADALL study that found that exposure to allergenic foods from 3 months of age reduced FA at 36 months in a general population (14). So far, management of food allergies follows two major strategies: focusing the initial symptoms or long-term treatments (15). One of the main strategies against FAs is the avoidance of allergen ingestion or contact; however, around 11% of allergic reactions are caused due to non-accidental contacts (7). A correct diagnosis of FA is key for the avoidance of allergic reactions; nowadays, the gold standard for food allergy diagnosis is the Oral Food Challenge, but other methods are also used, including blood IgE level measurements, Skin Prick Tests, physical examination, and anamnesis (16, 17). Most common allergic components of the diet are cow's milk, tree nuts, peanut, shellfish and vegetables, as determined by the US National Health and Nutrition Examination Survey (NHANES) (18).

Atopic dermatitis is the main inflammatory skin disease, whose prevalence reaches 20% in children and 3% in adults (19) producing several alterations of life quality. AD is driven by various pathophysiological mechanisms, including genetic factors, and is related to other diseases such as asthma, allergic rhinitis, and food allergy (20–22). In fact, AD is considered by several authors as the first cause that could trigger subsequent allergenic processes. The mechanisms of AD are multifactorial, involving barrier dysfunction and Th2 response, with a main role of IL-4 and IL-13, but also IL-31, IL-17, IL-22 and TSLP (23). Two types of AD have been described: the extrinsic form, with an IgE-mediation; and the intrinsic form, not IgE mediated (19). AD patients often manifest eczema with excoriation and serous exudation, and the treatment is based on corticosteroids, cyclosporine for dupilumab for patients that do not respond to topical therapies (24).

Mechanistically, allergic reaction development starts with the recognition of allergens by the immune system. Under certain conditions, this recognition triggers a reaction called sensitization phase_,_ that primes naïve B cells into differentiating plasma cells, that finally produce specific IgE against the allergen. As stated by “the atopic march theory”, skin barrier impairment during infancy and the development of AD acts as key drivers for further development of asthma or FAs due to cytokine release and defective barrier function (25). Even though not shared by all the allergic patients, a common feature is the disruption of an epithelial barrier during allergy development, mainly those from skin, gut, and lungs, matching the three more prevalent allergic diseases. The epithelial barriers constitute the main surfaces of contact between the inside of the body and the outside world and are strongly involved in the outcome of the immune responses, playing an active role in the sensitization phase during allergy development. In addition, other components in close contact with these barriers, such as the microbiome, have been proven to play key roles in epithelia homeostasis whereas dysbiosis may trigger a tolerance breakdown.

The most common manifestation of allergic responses to food involves the gut, but it is increasingly evident that gastrointestinal allergy to ingested foods often precedes or coexists with respiratory tract symptoms, rhinorrhea, sneezing, coughing, and wheezing. Asthmatics and patients with allergic rhinitis are often affected by gastrointestinal disorders (26). In addition, it has been shown that exposure to aeroallergens (e.g., pollen derived allergens such as profilin) not only have a significant effect on the development of allergic diseases in the lung but can also induce sensitization towards specific foods components (27). The opposite direction has been also shown in mice, as experimental gastrointestinal allergy to egg ovalbumin (OVA) enhances pulmonary responses to OVA but also to an unrelated allergen as house dust mite (28). As an opposite effect, evidence from mice has shown that dietary fiber, metabolized by a “healthy” gut microbiota, exerts a beneficial effect in the immunological environment in the lung protecting against the development of allergy and asthma (29, 30). Intestinal microbiota is of special interest as producer of fiber-derived short-chain fatty acids (SCFAs) that present beneficial effects not only in the gut but also in distant organs. Besides, SCFAs have a powerful anti-inflammatory effect and enhance epithelial function (31, 32). Taking all these considerations into account and in view of the highly interesting crosstalk between the gut microbiota and the lung epithelia in the context of allergy, we focused this review firstly on factors that modulate the integrity of epithelial barriers to focus finally on the role of gut derived SCFAs in the regulation of lung epithelia homeostasis.

## Epithelial barriers play a key role in allergy development

The different epithelial barriers of the human body constitute the first line of defense against harming agents, pathogens, or even allergens. Growing evidence supports the “epithelial barrier hypothesis”, which states that epithelial barrier integrity and function is key to maintain the homeostasis of the organism and that a dysfunctional barrier may underlie allergic and other inflammatory diseases and explain their increase in prevalence (33–35).

**Figure 1:**
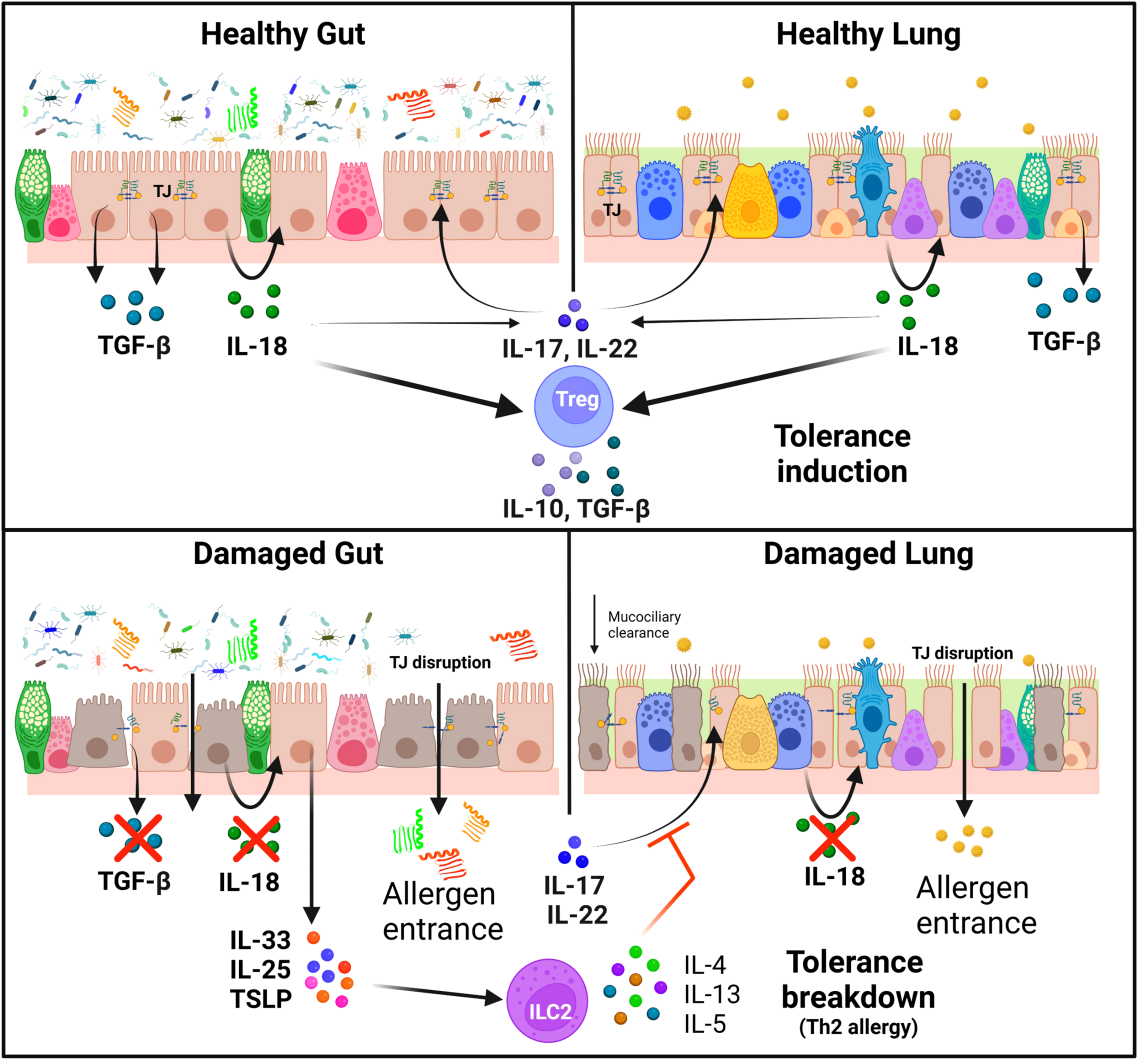
Immunoregulation of tolerance induction and breakdown in healthy and damaged epithelia. Tight junctions are essential for the maintenance of healthy gut and lung epithelial barriers. IL-17 and IL-22 induce the expression of TJ proteins, but this effect is impaired in the presence of Th2 cytokines. Healthy epithelial cells release IL-18 and TGF-β in response to allergens, which enhance the production of IL-17 and IL-22 and stimulate T_reg_ cells to release IL-10 and TGF-β, leading to tolerance induction. On the other hand, epithelial cells in damaged barriers secrete alarmins (IL-25, IL-33 and TSLP) which trigger tolerance breakdown with the activation of a Th2 response in ILC2 cells with the release of IL4, IL-5 and IL-13 (Created with BioRender.com).

Even though each epithelia have a different role and morphology, they share many physiological and structural/mechanical features. The apical junction complexes (AJCs) are one of the main components maintaining the integrity of epithelial barriers as they regulate cell-cell adhesion, cell polarity, and paracellular permeability of exogenous elements. These complexes are composed of two main structures, the tight junctions (TJs) and the *adherens* junctions (AJs) (36–38). TJs are formed by extracellular domains of transmembrane proteins (such as occludin and claudin protein families) that form strong links between them and connect with actin and tubulin cytoskeleton *via* scaffold proteins such as *zonula occludens*-1 (ZO-1) (39). TJ formation is regulated by the expression and phosphorylation of their components or the expression of disruptor proteins (e.g., Claudin 2) (40). Allergen tolerance is directly related to TJs integrity: IL-17 and IL-22 induce ZO-1 and claudin expression; but, in atopic individuals, this route is impaired because of the presence of Th2 cytokines facilitating the entrance of allergens (41, 42) (Figure 1).

Epithelial cells have indeed an immunologic function: they are the first sensors of external damage, producing and secreting alarmins IL-25, IL-33 and thymic stromal lymphopoietin (TSLP), triggering the activation of type 2 innate lymphoid cells (ILC2) Th2 responses (33, 43). This activation is key for sensitization and in the physiopathology of allergic diseases, triggering epithelial damage and an increase in its permeability. ILC2 cells regulate and crosstalk with naïve T cells, showing an important role for specific CD4^+^ T cell production by secreting IL-5 and IL-13. They participate on IL-4 mediated Th2 differentiation, and promote mast cell sensibility to degranulation, but also inhibit T_reg_ induction (44). They also have a higher cytokine production rate in comparison to Th2 when activated and have the capacity of antigen presentation *via* MHCII (45).

Thus, these epithelial cell-derived cytokines can be considered as novel targets for allergy treatment (46). Currently, an anti-TSLP monoclonal antibody has been approved in the US for the treatment of uncontrolled severe asthma (47), and an anti-IL-33 is being proved in clinical trials against AD (48) and peanut allergy (49). Moreover, intestinal epithelial cells (IECs) express MHCI/II, thus having the possibility to induce specific antigen immune responses towards allergenic components, and these cells secrete exosomes containing these MHCII-peptide complexes mediating indirect presentation of allergens that prime for an immunogenic rather than tolerogenic response (50, 51).

Healthy epithelia have also a regulatory function: they sense the environment and activate different routes of homeostatic responses *via* toll-like receptors (TLR), protease-activated receptors (PAR) and nucleotide-binding leucine-rich repeat-containing receptors (NLR) (52–54). The gut epithelia also secrete antimicrobial peptides (AMPs) that play an important role in maintaining tolerance to gut microbiota, protecting against enteric infections, and thus maintaining a healthy microbiome. In addition, some AMPs present anti-inflammatory and immunostimulatory properties. For example, human α/β-defensins and cathelicidin regulate the intestinal microbiota, limiting invasion of the epithelia and acting against gram-positive commensals, restricting enterohemorrhagic *E. coli* infections; lactoferrin and hepticidin control free iron required for bacterial growth and lysozyme enzymatically degrades the peptidoglycan of gram-positive bacteria inducing their lysis (55, 56).

Inflammatory responses also alter intracellular signaling routes that are intrinsically linked to the maintenance of the barrier integrity (57) (Figure 1). Classic Th2 cytokines IL-4 and IL-13 contribute to TJ instability in skin and the lungs (58). Moreover, IL-4, IL-5, and IL-13 signaling cascades can be triggered after the cell response to barrier-disrupting noxious stimuli (33). In addition, other inflammation-derived molecules, such as IL-6 (57) or histamine (59), are known to induce epithelial barrier permeability.

## Environmental factors promote allergic diseases by disrupting epithelial barriers

Western lifestyle, diet, and environment account among the more commonly described factors contributing to the development of the allergic diseases. Humans are daily exposed to a plethora of different chemical and biological agents, overall known as the exposome (60). Human exposome includes compounds related with pollution (61, 62), hygiene-derived products such as laundry detergents (63), house dust mites (64), natural toxins (e.g., the mycotoxin deoxynivanelol) (65), and food additives (e.g., food emulsifiers) (66), which are mostly harmful for highly exposed body cell surfaces like the epithelial barriers. Several *in vitro* studies show that when the human epithelial barrier is exposed to these compounds, cell functions are altered (67, 68), and allergenic responses could be enhanced (69–74). Moreover, an adverse outcome pathway (AOP) describing the covalent binding of electrophilic chemicals to keratinocyte proteins leading to skin sensitization has been described (https://aopwiki.org/aops/40). This AOP is quite interesting as the covalent binding of electrophiles could potentially affect proteins from epithelial cells form other organs triggering their activation, leading to further activation of dendritic cells and T-cells and sensitization towards these electrophilic compounds.

Diverse environmental proteases have been described as allergenic initiators triggering further immune responses. Among them, it is important to mention those from house mite feces (whose presence in the exposome is directly related with western lifestyle), such as Der p 1 (75), and fungal proteases, such as Asp f 13 (76). The activity of these proteases disrupts the human TJs and increases the epithelial barrier permeability, facilitating the passage of diverse allergens according to *in vitro* studies (74, 77), and the stimulation of the innate immune system (78, 79). Their effect is regulated by the redox microenvironment of the bronchial lumen, as it has been described that Der p 1 activity is enhanced by glutathione-*S*-transferase-pi, a detox enzyme secreted by the bronchial epithelium, and by the presence of the antioxidant glutathione, both of which are highly abundant in the human epithelial lung fluid (80). This effect is stronger on damaged or inflamed epithelium (as occurs in asthmatic patients), in which anti-protease and mucociliary clearance are impaired (81). Protease inhalation is also related to FA, as a higher response to food allergens and an associated gut epithelium disfunction has been described in mice after intranasal exposure to dust mite extracts (82).

Environmental proteases as Der p 1 not only affect human bronchial epithelium, but also alter the integrity of intestinal epithelium as they are present in the diet together with food derived allergens with protease activity (e.g., Act d 1, a Cys-protease from kiwifruit) (83, 84). Der p 1 has been detected on human intestinal biopsies, where it not only disrupts the epithelial barrier integrity but also reduces the expression of TJ proteins and mucus barrier and induces a pro-inflammatory response with increased cytokine release (85, 86). Apart from barrier disruption, it is worth mentioning that alternative mechanisms could be acting at promoting allergic sensitization triggered by airborne proteases. In this sense, and although much less explored, Der p 1 cleavage of other secondary allergens have been described to generate small and more permeable allergen-derived peptides, which indeed preserve IgG/IgE reactivity and activation of basophils from allergic patients (77).

Several other exposome components of the urban environment produce or synergistically enhance epithelial damage, as has been shown in asthma and allergic rhinitis patients (87). On the inhaled group of toxic agents, it is important to mention traffic and industry related contamination (NO_2_, O_3_, particles in suspension), whose levels have been related geographically with a higher rate of infant asthma (88). Maternal exposure to NO_2_ causes an impairment in Th1/Th2 balance in newborn mice, leading to enhanced sensitivity to allergens and increased airway hyperresponsiveness (89). Increased Th2 response and accumulation of ILC2 cells was observed in a diesel exhaust-enhanced allergic mice model (90). Ozone and NO_2_ have, as well, a pro-inflammatory effect upon bronchial epithelial cells, promoting the release of cytokines and chemokines, such as IL-33, IL-25 and TSLP, in both normal and asthmatic patients (91, 92).

Tobacco smoke exposure also exacerbates asthma and rhinitis symptoms and decreases muco-ciliary clearance (93). Moreover, there is a strong epidemiological link between pre- or postnatal passive cigarette smoke exposure and the prevalence of asthma in children (94, 95). Recent studies have spotlighted the effect of cigarette smoke on the pulmonary epithelium, directly disrupting TJs and modulating TJ protein expression and aggravating OVA-induced inflammation in asthmatic mice models (96).

Cigarette smoke exposure also modifies epigenetic marks involved in immune response, such as increasing methylation of GC isles in genes like IL-10 in human samples (97) and promote Th2 responses, e.g., by decreasing gene methylation of IL-4, IL-13 or increasing FOXP3 methylation after house dust mite challenge (44).

Other components from the exposome can also alter the pulmonary epithelium and facilitate the induction of respiratory allergic diseases. On this idea, it has been reported that inhalation of airborne microplastics causes pulmonary inflammatory cell infiltration, bronchoalveolar macrophage aggregation and increased levels of tumor necrosis factor-α (TNF-α) in both healthy and asthmatic mice (98), and that viral infections during infancy and childhood predispose to later asthma development (99, 100).

## Diet modulates epithelial integrity: protective and harmful food factors

Perinatal and early age nutrition, and the correct time of active introduction of dietary components is key to avoid FAs and the development of tolerance. The actual tendency is to potentiate this development by introducing probiotics and lactose in child formulas (101). As described above, a damaged gut epithelium is related to the development not only of FAs but also of other types of allergies. In fact, there is increasing evidence of the presence of ingested noxious agents in the exposome that contribute to this damage. During the early life introduction of different foods, especially when breastfeeding is being retired (or has been absent), the child is lacking the protective compounds supplied by mother's milk. In this context, some foods and dietary components, especially from fish and vegetable meals, have been proven to possess a protective effect against gut epithelium damage and even in distant epithelia such as the lung, either directly or after intestinal microbiome processing (102–104).

**Figure 2:**
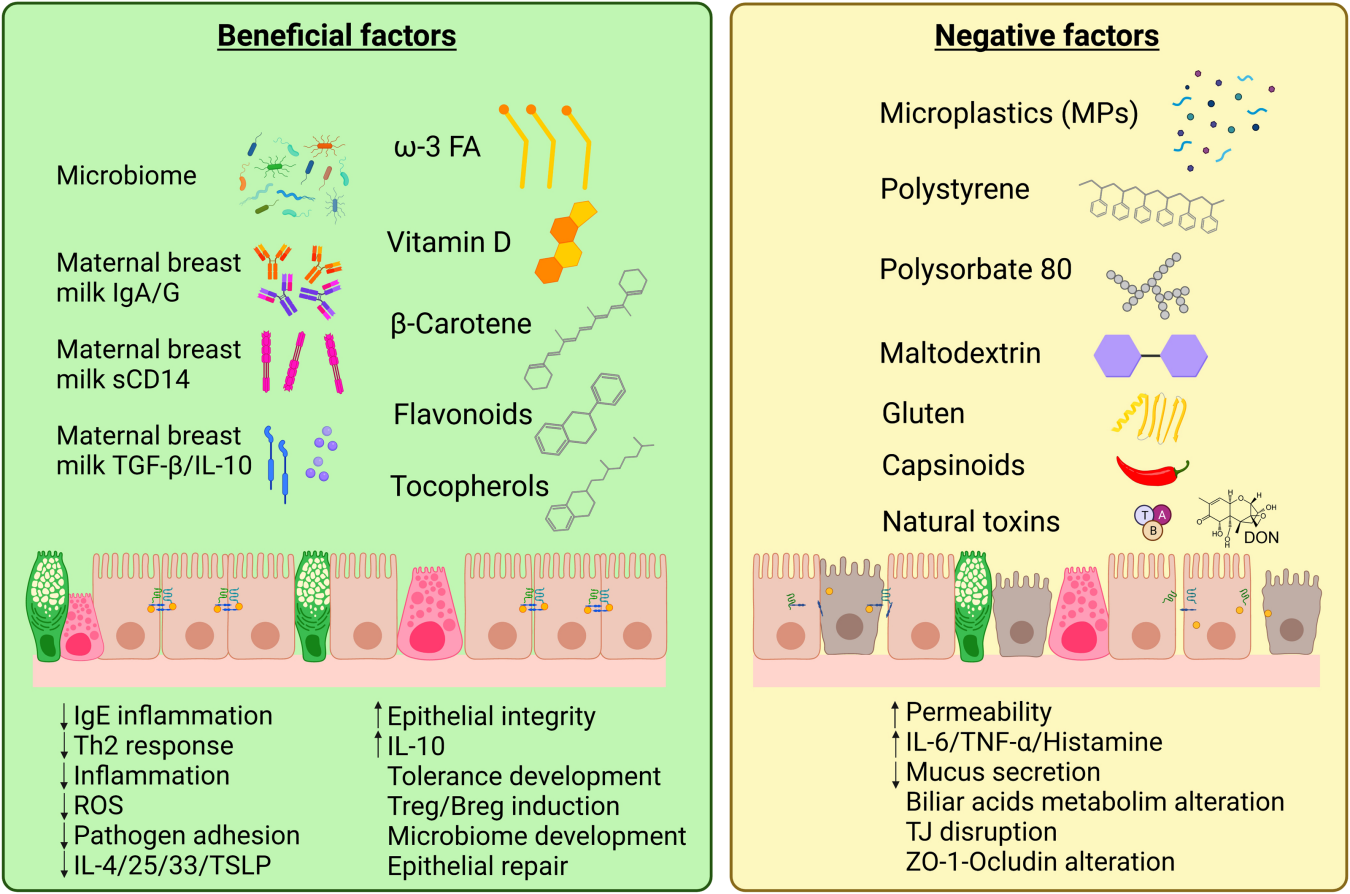
Diet components that present beneficial or negative effects on the intestinal epithelial barrier. An equilibrated diet and maternal breast milk contains a wide variety of beneficial factors that enhance intestinal epithelium integrity and repair. However, western lifestyle and pollution have introduced new agents in our diet that, together with natural compounds as gluten, capsinoids or natural toxins [e.g., mycotoxins as deoxynivanelol (DON)], can be irritant or present negative effects on gut epithelial cells increasing barrier permeability and potentially triggering allergic responses (Created with BioRender.com).

Even though some diet components and contaminants may alter the epithelial barriers, there are several other diet components that present protective effects against allergic diseases by reinforcing the epithelial barrier (Figure 2). EAACI 2020 guidelines point towards the beneficial effect of breastfeeding by preventing the development of food allergy in the first two years of life, or even against asthma and allergic rhinitis (105, 106). Maternal milk contains a milieu of beneficial components; however, their levels vary between individuals, especially when comparing atopic mothers with non-atopic mothers. Maternal secreted IgA is directly able to raise the infant immune system as it protects from respiratory and digestive tract infections, thus preserving epithelial integrity. Lower levels of IgA in milk are related to higher risk of cow milk allergy (107). Soluble CD14, that is produced by mammary epithelial cells, is another powerful immunomodulatory molecule; it mediates secretion of innate immune response molecules such as IL-8, TNF-α, and epithelial neutrophil activator-78 by CD14-negative intestinal epithelial cells exposed to lipopolysaccharide (LPS) or bacteria (108). CD14 acts as a co-receptor for TLR4 and enhances the recognition of bacterial LPS by the immune system, especially at low LPS levels (109). This CD14-TLR4 interaction plays a key role in immune tolerance development during early life, as evidenced in the correlation between low CD14 levels in breast milk with higher risk of AD (110) and sensitization to egg white (111). Breast milk also contains TGF-β, a powerful anti-inflammatory cytokine that is also involved in gut epithelial integrity, IL-10, an inducer of antigen-specific tolerogenic T_reg_ and B_reg_ cells, and IL-6, a pro-inflammatory cytokine that stimulates Th-17 responses in the presence of IL-10 and TGF-β (112, 113). These molecules are absent or undetectable in substitutive formula for breastmilk.

Early sensitization to food allergens during the breastfeeding, or even in the amniotic fluid, explain reactions to a food with which there has been no previous contact. In fact, the presence of major food allergens in breast milk and amniotic fluid has been shown in the range of nanograms per milliliter, as described for OVA, β-lactoglobulin from cow and sheep's milk, peanut proteins Ara h 1/2/6, among others from fruit, mustard, wheat or fish (114–116). The kinetics of antigen shedding and clearance strongly depend on the mother's diet, but varies with individuals, the way of antigen consumption (i.e., raw or cooked), or with the interaction of other milk components (115). Specific IgG from an allergen-exposed non-atopic mother is related to the onset of antigen-specific tolerance in the infant due to the formation of IgG-antigen immune complexes in breast milk that potentiate specific T_reg_ cell development (117, 118).

Human breast milk also contains a lipid fraction, which is responsible for many beneficial properties. Special attention has been paid to polyunsaturated fatty acids (PUFAs), mainly ω-3 fatty acids like eicosapentanoic (EPA) and docosahexanoic (DHA) acids, that depend directly on the mother's diet (e.g., fish oil supplementation) and are currently under study due to the correlation of breast milk lipids and the risk of allergic disease (119, 120). Oligosaccharides from breast milk are additional agents that potentiate epithelial barrier function and protect against pathogen adhesion. Moreover, they contribute to the early life microbiota development (as will be discussed later in this review) (121).

In addition to their presence in breast milk, ω-3 long-chain PUFAs (DHA and EPA) are present in various food sources and can be incorporated in the routine diet. As described above, they play a key role in immune system development and in the establishment of tolerance by suppressing inflammation (122). These two PUFAs, together with ω-6 arachidonic acid (AA), are widely present in immune cell membranes. Free AA is oxidated *via* cyclooxygenase (COX) and lipoxygenase (LOX) pathways, releasing prostaglandins and leukotrienes. The role of AA-derived metabolites in allergic disease is still controversial. In one hand, they enhance inflammation, histamine production, white cell infiltration in epithelia and suppress Th1 inflammation; on the other hand, prostaglandin E2 is reduced in severe anaphylactic patients' sera (123). DHA and EPA suppress AA-mediated inflammation as their oxidation produces anti-inflammatory molecules. In fact, both PUFAs from fish oil supplements have been reported to ameliorate allergic responses in children with risk of developing allergy (124). Novel roles for these acids have been also described in *in vitro* porcine and human models, as they interact with PPAR-α and PPAR-γ in intestinal epithelial cells, enhancing epithelial cell function (125), protecting against exogenous damage (126) and decreasing cytokine-mediated permeability (127). High DHA diet al.so enhances repair of dust-exposed pulmonary epithelium in mice (128).

Vitamin D is a fat-soluble sterol whose deficiency has been related to immune deficiencies and is also involved in the regulation of gut microbiota. Humans can synthesize it after sunlight exposure (which is reduced by western lifestyle) or obtain it from animal- and plant-derived food. Vitamin D contributes to intestinal homeostasis inducing the expression of the antimicrobial peptide cathelicidin in IECs (129) and is essential to maintain the integrity of the gut mucosal barrier by enhancement of intercellular junctions that control mucosal permeability and reduction of pro-inflammatory cytokines such as IL-8; therefore, its deficiency is related to a leaky gut (130, 131). It also has a potent tolerogenic effect towards the immune system, inducing IL-10 secretion and contributing to T_reg_ differentiation (132).

Nevertheless, there are discrepancies as to the potential benefits of supplementing diet with vitamin D regarding the prevention of allergic diseases; these beneficial effects are not clear in FAs and AD (133) and only some studies detect a decrease in the incidence of asthma and rhinitis (134). Prenatal administration to pregnant woman with asthma risky child induces a slight descent on the 3-year incidence of asthma and rhinitis, but did not influence the 6-year incidence (135). Vitamin D supplementation, combined with immunotherapy, enhances FoxP3 expression on specific therapy towards AD according to human *in vivo* studies (136); however, no symptomatologic differences were detected compared to patients treated with immunotherapy alone. On a cohort of Icelander children that received (or not) vitamin D supplementation, this administration correlated with a 6-year lower risk of allergic sensitization (137). But vitamin D administration is inefficient on asthmatic patients in which the vitamin blood levels are already low (138). This may correlate with an increase in oxidative stress in the gut due to pollutants and increased luminal levels of oxidized vitamin D metabolites that do not bind to the vitamin D receptors (139). Low vitamin D blood levels can be also due to malabsorption of the vitamin, as has been reported in several cases of food allergies (140). Thus, vitamin D diet supplementation may not be effective in these patients. In summary, authors conclude that enough vitamin D administration is key for tolerance development (specially on Nordic countries with limited sunlight), but the beneficial effect is still controversial.

Dietary antioxidants have been associated with a protective effect against allergic diseases (141). Diverse vitamins and vegetable-derived compounds have been proven to exert beneficial effects on human health, given the fact that oxidative stress impairs the gut metabolism of Vitamin D (139). Liposoluble vitamins β-carotene (pro-vitamin A) and tocopherol (vitamin E), obtained only from fresh vegetables and nuts, have also proven their protective effects against allergy due to their strong antioxidant properties. Vitamin E impairs Th2 inflammation on mice models, inhibiting eosinophil and neutrophil activation and the production of oxygen reactive species (142). Intranasal administration of tocopherol is a current approach to ameliorate symptoms in allergic rhinitis patients, but oral supplementation is not proven to be efficient in AD, FA or asthma treatment (143).

Plant flavonoids like kaempferol or quercetin are also known as inflammation suppressors with increasing relevance in the field of allergy. Both reduce IgE-induced inflammation in human IECs (144) and kaempferol mitigates inflammation in asthma models when orally administrated to OVA-sensitized mice (145). *In vivo* studies showed that quercetin ameliorates mice epithelial asthmatic response inhibiting the secretion of IL-4, IL-25, IL-33 and TSLP and leads to lower mast cell infiltration and endothelial smooth muscle thickness (146).

Diet can also contain harmful components or additives that may impair the intestinal epithelial barrier function and thus foster inflammatory diseases, such as inflammatory bowel disease, obesity and celiac disease (33, 147), as well as FA, where the allergens can cross the leaky epithelial barrier and reach immune cells (148) (Figure 2). Along with lifestyle changes and pollution increase, dietary changes have occurred specially in urban environments, with reduced fresh food intake and increased processed and junk foods.

Food additives, mainly dietary surfactants, are on the focus due to their damaging properties for the epithelial barrier showing toxic and gut permeabilizing effects (149). For example, the food emulsifier Polysorbate-80 has been reported to increase the permeability to food allergens in rodents and human IECs (150–152), as well as presenting effects on distant tissues, being able to alter the systemic metabolism leading to glucose intolerance and mitochondrial dysfunction in the skeletal muscle in mice (153). Maltodextrin, an alimentary thickener widely used in infant formula, induces reticular stress, inflammation, and mucin deficit on the intestinal epithelium (154). In addition to food additives, other diet components can also alter the epithelial barrier status. For example, gliadin can alter the interactions between occludins and ZO-1 on humans (155), or hot spices components (such as capsianosides and terpene glycosides) alter TJ integrity and the paracellular flux by affecting the actin cytoskeleton (156, 157).

Because of environmental contamination, and due to the high recalcitrance of many synthetic plastics that result in their long persistence in the environment, small plastic particles (microplastics or MPs) have been incorporated to our diets. These MPs accumulate in the marine ecosystem, being ingested by invertebrates and fish, and can also leak into soil and accumulate in plants, thus entering the trophic chain being an increasing matter of concern in human health (158, 159). As an example, polystyrene MP have been shown to affect directly epithelial permeability and alter biliary acid metabolism in mice (160). Other studies have related the ingestion of these MPs with mucus secretion, glucose and lipid metabolism, and microbiota alterations (161). Furthermore, polypropylene MPs have been associated with immune system alterations (162), inducing an increase in the secretion of pro-inflammatory cytokines (e.g., IL-6 and TNF-α) and histamine in human and mice cells *in vitro* (163). Interestingly, most of the damage caused by MPs on the intestinal barrier seem to be more directly dependent on the particle size and not so much on their composition (163, 164). Altogether, these data point towards a potential contribution of MPs to allergic diseases development due to the deleterious effect on the gut epithelium and their pro-inflammatory potential (68, 165); nevertheless, further research is required to clarify MP effects in human health.

## Gut microbiome as a key regulator of the intestinal epithelial barrier status and immune response

**Figure 3:**
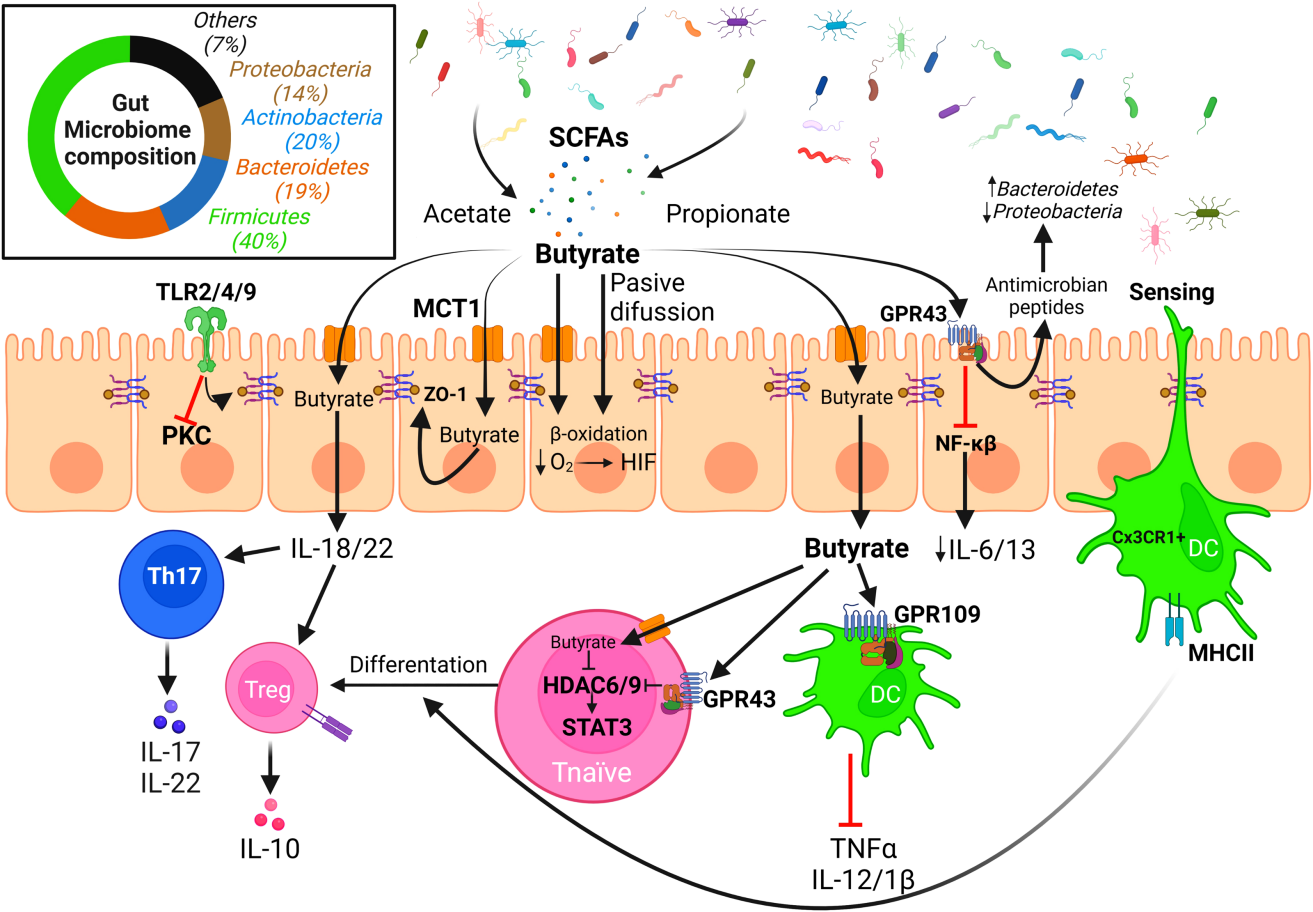
Effect of microbiota derived SCFAs on the integrity of the intestinal epithelial barrier. The intestinal microbiota is mainly composed of bacteria from the phyla *Firmicutes/Bacillota, Actinobacteria*, *Bacteroidetes*, and *Proteobacteria/Pseudomonadota* among other minor phyla. Diet fiber fermentation by gut microbiota generates SCFAs that may enter the epithelial cells either through passive diffusion or *via* monocarboxylate transporters, or well interact with GPCRs (e.g., GPR43) present in the apical membrane. Butyrate is the main fuel and carbon source for colonocytes, but it can be exported through the basolateral membranes and interact with immune cells altering T cells differentiation and cytokine release patterns, promoting the integrity of the epithelial barrier. Biological effects of butyrate are mainly related to its activity as inhibitor of HDACs, effect that is enhanced *via* interaction with GPR43 that induces a down-regulation of HDAC expression (Created with BioRender.com).

It is increasingly evident that dysbiosis of the human commensal gut bacteria triggered by western lifestyle may contribute to food allergy (166–169). *In vitro/ex vivo* studies showed that gut microbiome is composed not only by bacteria, but also by other microorganisms including fungi, archaea, virus and protozoans (170). Among healthy individuals, the microbiota composition is quite similar, despite minor variations based on own individual diet and lifestyle (Figure 3). The main bacterial phyla present in the human healthy gut are *Firmicutes* (classes *Clostridia* and *Lactobacillus*)*, Bacteroidetes* (class *Bacteroidales*), *Actinobacteria* (class *Bifidobacteriaceae*), and *Pseudomonadota*/*Proteobacteria* (class *Enterobacterales*) (Figure 3) (171). However, important differences in microbiome species have been described in diseases such as inflammatory bowel disease (37, 170), celiac disease (172) or food allergies (173, 174), among others.

The development and maintenance of a healthy gut microbiome is key to human health status as it contributes to many physiological functions: it impairs pathogen colonization, promotes gut epithelial and mucosal integrity, produces necessary vitamins B12 and K, and has a potent immunomodulatory role (175). Even though the question of what a healthy microbiome is remains unsolved, some species have been related to human microbial health, specifically with allergic diseases. *Bacteroides fragillis* and *Bacteroides stercosis* are specially relevant species, even though they are only moderately abundant in the human gut, as they modulate the production of key metabolites that influence the composition of the microbiota (176). The biodiversity hypothesis states that microbiota species enrichment promotes immune tolerance, whereas low microbiome species diversity is associated with allergic diseases (177). However, the increase in certain bacterial populations, such as *Clostridium* or *Firmicutes,* are associated with an enhanced allergic sensitization risk in young children according to various cohort studies (178–180).

Homeostatic and beneficial properties of commensal bacteria are mediated by small metabolites that can cross epithelial barriers and exert their effects locally or in distant epithelia as the lung or the skin after entering the bloodstream, inducing cytokine release. Short-chain fatty acids (SCFAs), particularly butyrate, propionate and acetate, are among the main products of dietary fiber fermentation, with a total concentration in the human intestinal lumen decreasing from 70 to 140 mM in the proximal colon to 20–70 mM in the distant colon (181). These SCFAs are key players in the microbiome-immune system crosstalk (Figure 3) (182). They present beneficial effects regarding FAs; among them, they modulate gut epithelial cell function and barrier integrity (31, 32).

Butyrate is used as a preferential carbon and energy source by human IECs, being consumed by the β-oxidative pathway and shifting energy metabolism (183). Butyrate metabolism decreases available intracellular O_2_, leading to hypoxia induced factor (HIF) activation, which enhances gut integrity and is necessary for butyrate-mediated intestinal barrier restoration (184, 185). SCFAs are recognized by specific G protein-coupled receptors (GPCRs), such as GPR109 or GPR43, present on the surface of intestinal and immune cells. This interaction generates anti-sensitization responses on the epithelial barrier and induce T_reg_ cell differentiation in the colon (186–189). Depletion of these receptors provoke a disruption of the gut epithelial barrier on mouse models, increasing allergen permeability and inducing specific IgE reactions (190). GPR43 is expressed on a higher rate on epithelial cells rather than dendritic cells (DCs), whose main receptor is GPR109. GPR43 activation in IECs induces antimicrobial peptide production, which regulates microbiota composition by enhancing *Bacteroidetes* population and decreasing *Proteobacteria* proportion (191). GPR43^−/−^ mice had this effect reversed when butyrate was added to drinking water, accompanied by an increase of *Clostridium* and *Proteobacteria* (190, 191). GPR43 has a maximal effective concentration threshold in human epithelial cells of around 0.5 mM, and its activation triggers downstream signaling that provokes cAMP decrease and increases cytoplasmic calcium concentration (192). This receptor induces also an alternative anti-inflammatory signaling mediated by β-arrestin-2, by inhibition of the NF-κB pathway, and down-regulation of pro-inflammatory cytokines IL-6 and IL-1β (193).

*In vivo* experiments revealed that high fiber diets, that increase butyrate production in the gut, enhance the expression of cellular adhesion proteins such as occludin and ZO-1, and induce IL-22 expression, thus contributing to the integrity of the gut mucosal barrier while reducing the concentration of pro-inflammatory IL-21 (189). SCFAs, mainly butyrate, induce the production of IL-18 in IECs (194), an additional key cytokine for the maintenance of barrier homeostasis (195). TSLP, that polarizes dendritic cells (DCs) to adopt a CD103-(+) Th2 phenotype, is enhanced in GPR43^−/−^ mice, but paradoxically not altered when GPR109 is absent (190). In addition, SCFAs restore T_reg_ numbers in mice devoid of a gut microbiota and regulate T_reg_ function *via* GPR43 inducing a reduced expression of histones deacetylases (HDACs) 6 and 9 (196).

Bacterial-derived SFCAs, mainly butyrate, also regulate the epigenetic status of the human epithelium by inhibition of HDAC activities, specifically zinc-dependent class Ia and IIa HDACs (197). Butyrate enters epithelial and immune cells *via* passive diffusion or through different monocarboxylate transporters as MCT1 or a butyrate/bicarbonate antiporter (183). This leads to HDAC inhibition (together with the above mentioned down-regulation of the expression of some HDACs *via* GPR43), causing an opening of the chromatin and facilitating the binding of STAT3, NF-κB or Foxp3, important factors on the development and regulation of immune cells (193). *In vitro* studies demonstrated that this provokes a repressed production of pro-inflammatory molecules TNFα, IL-12, IL-1β, and NO on immune cells, while upregulating anti-inflammatory IL-10 production on mononuclear cells and neutrophils (193). Once butyrate enters the cell, it also has the capability of activating PPAR-γ (198), promoting epithelial barrier integrity (199).

Other SCFAs such as acetate and propionate have also some effects on immune responses, increasing the expression of IFN-β in the lungs *via* GPR43 activation (200) or inducing CD69 expression on basophils and IL-13 production (201), but their role in allergy seems to be less relevant than that of butyrate.

The microbiome, either through its metabolites or through direct interaction with cells, influences immune responses, with the epithelial barrier as central element of transduction. SCFAs induce different cytokine secretion by gut epithelia, as we have previously reviewed, which modulate the immune system response. Human IECs express on their surface TLR2/4/9, that can recognize exogenous elements present on the lumen. Activation of TLR2 enhances ZO-1 expression in human IECs *via* activation of PKC, whereas TLR4 activation reduces this expression (202). IECs can also produce and secrete several types of cytokines under different stimuli. For example, butyrate induces *in vivo* IL-18 secretion by mice IECs modulating Th17 and T_reg_ cells, controlling their differentiation (203). In response to allergen exposure, IECs can produce IL-25, IL-33 and TSLP and signal to ILC2 cells, that respond secreting Th2 cytokines (IL-4, IL5, IL-13), and IL-9 (204–206). These cytokines have the ability of inducing differentiation of progenitor cells to secretory cells, perpetuating allergic inflammation (207). Together with IL-9, IECs can also secrete eotaxin-1 that recruit eosinophils to the gut when an allergen is present (208). Otherwise, IL-22 secreted from immune cells, such as Th1/17, affects the state of the epithelial barrier, inducing cell survival *via* secretion of antimicrobial peptides from the Reg family (RegIIIβ and RegIIIγ) in colonic epithelial cells (209).

The immune system has also mechanisms to directly sense the microbiome and antigens which are present in the lumen. CX3CR1^+^ DCs generate protrusions that reach the lumen, crossing epithelial cell contacts, and capture antigens directly without epithelial processing preserving the integrity of the epithelial barrier (210, 211). These DCs express CD11c and CD103, *via* TGF-β and retinoic acid produced by IECs, promote T_reg_ differentiation (212), and induce integrin α_4_β_7_ expression which is involved in gut homing of T_reg_ cells (213, 214).

## Microbial and dietary regulation of the pulmonary epithelium: the gut-lung axis

Despite their manifest anatomical distance, alteration of the human microbiome resident in the gut has been linked to inflammatory conditions on the airways mainly *via* SCFAs (acetate, propionate and butyrate) derived from bacterial fermentation of diet fiber (215), contributing to the definition of a novel player in allergic diseases: the gut-lung axis (216). SCFAs and small bacterial metabolites have a positive effect as well in different epithelia along the body, contributing significantly to the skin barrier function (defining the gut-skin axis) by modulating keratinocyte metabolism in fiber-fed mice models (217) and even to the esophageal barrier function *in vitro* (218).

There is increasing evidence of the influence of intestinal microbiota metabolites in the maintenance of lung epithelium homeostasis and integrity. Cohort studies have shown a systematic decrease in SCFA-producing *Bifidobacteria* in long-term asthmatic patients, spotlighting the relevance of *Bifidobacterium adolescentis* and *Bifidobacterium breve* as inflammation suppressors (219, 220). In a similar way, a decrease of *Akkermnansia* and *Faecalibacteria* species in the human gut lumen has been related *in vivo* to the development of asthma and atopy in parallel with an alteration on the fiber fermentation and SCFAs production (221). These results are also supported by the European PASTURE/EFRAIM study group, that has reported that high concentrations of fecal butyrate and propionate in children at the age of one year had significantly less atopic sensitization and were less susceptible to develop asthma between 3 and 6 years, together with a reduced risk of food allergy and allergic rhinitis (222). SCFAs have a protective role against allergic sensitization in the lungs, as they are able to downregulate hematopoiesis during Th2 allergic airway inflammation (30), enhancing macrophage and DC progenitors, that later infiltrate in the lungs and mature to CD11b^+^ DCs which are unable to present allergens (223, 224). *In vivo* studies with butyrate oral administration demonstrated an attenuation of OVA-induced asthma and lung infiltration of eosinophils into lungs on mice (222). Similar results were observed with high fiber fed mice challenged with house-dust mite (29).

On the contrary, some bacterial species from the genus *Clostridium* have been typically associated with pro-inflammatory roles (e.g., pathogenic *Clostridia* or *Clostridium neonatale*) and they are increased in stool samples from asthmatic children related to controls (225). However, *Clostridium butyricum* has been recently associated with tolerance development (226).

**Figure 4:**
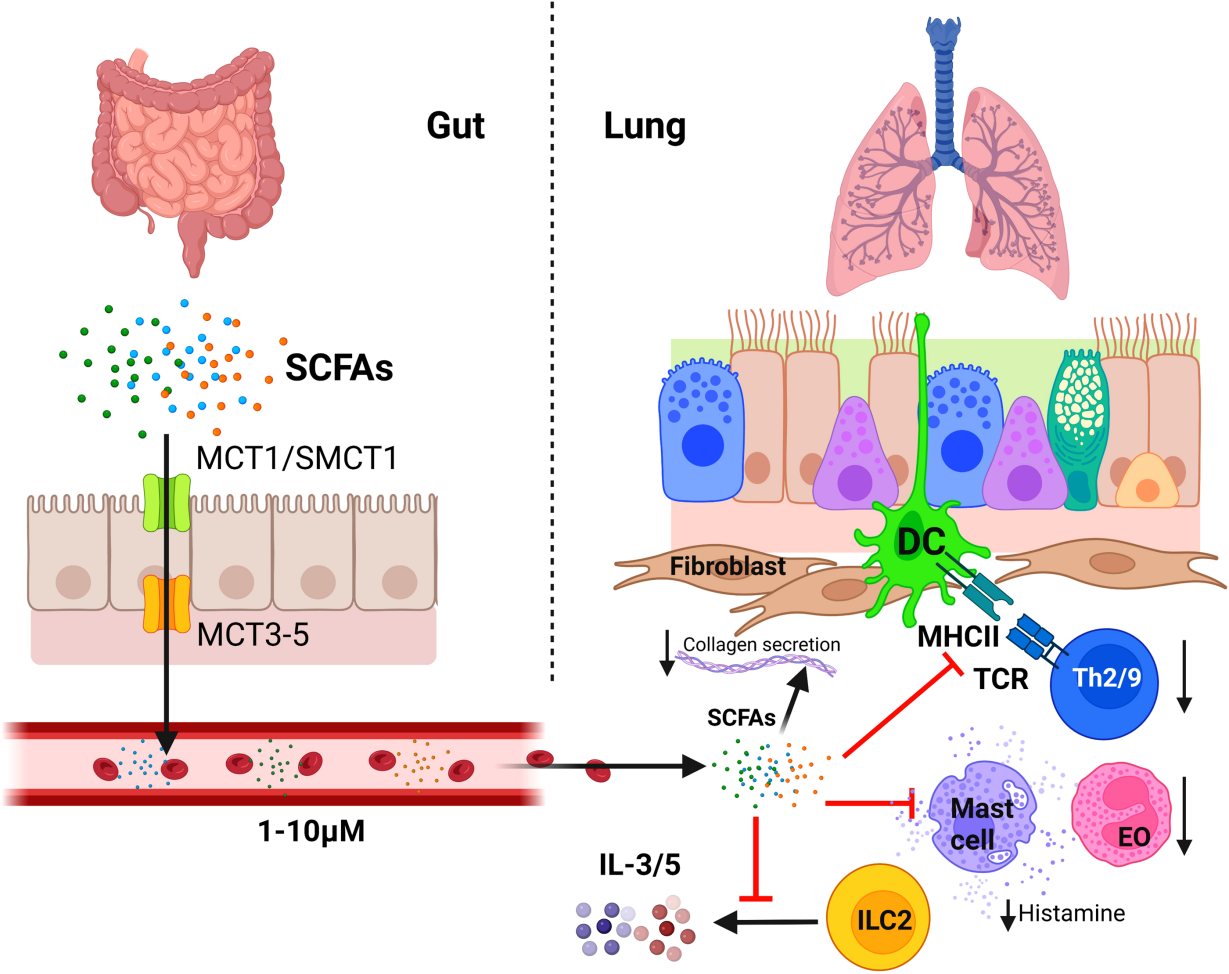
Regulatory influence of the gastrointestinal microbiota on the immunology of the lung: the gut-lung axis. Butyrate, propionate, and acetate produced by gut microbiota cross the intestinal epithelial barrier through monocarboxylate transporters and reach distant organs through the bloodstream and the lymphatic circulatory system. In the lung, SCFAs affect the bronchial-associated lymphoid tissue acting as efficient suppressors of the allergic immune reaction. Among other effects, they induce an inhibition of Th2 cell activation and induce T_reg_ cell differentiation, inhibit IL-3 and IL-5 secretion by ILC2 cells and the migration of eosinophils (EO) to airways, induces eosinophils apoptosis, and inhibits mast cell degranulation. In addition, SCFAs can also reduce allergic bronchoconstriction and pulmonary fibrosis by inducing a down-regulation of collagen synthesis by lung fibroblasts (Created with BioRender.com).

Even though SCFAs play a key role in tolerance to aeroallergens and pulmonary inflammatory status, lung microbiome cannot produce them, being specifically secreted by some populations of the gut microbiome establishing a unique relationship between diet, gut microbiome, and respiratory allergy. Microbiome-produced or diet-derived butyrate enters the bloodstream crossing the intestinal epithelial barrier *via* apical surface MCT1 or SMCT1 transporter proteins in the epithelial cells (227); although butyrate is used by colonocytes as main energy and carbon source, it can still be exported through the basolateral membranes *via* MCT3–5 (228) reaching the bloodstream (Figure 4). Propionate and butyrate are metabolized in the liver, but they can still reach peripheral organs, such as the lungs or the bone marrow, where concentration of these SCFAs is directly correlated with fiber consumption (229). High-fiber diet is known to cause a 20-fold increase in butyrate concentration in portal blood and a 6-fold increase in arterial blood in porcine models (230), and 100-fold in venous blood in murine models (231). Butyrate and propionate concentration in human plasma is around 1–10 μM (229); however, there are no clear studies on the effect of diet or microbiota in SCFA concentrations in human peripheral blood due to limited accessibility of blood samples and the requirement of complex quantification techniques, such as mass spectrometry or heavy isotope labeling. In any case, new and simpler methods for determining SCFAs in human blood are currently under development with potential application in this field (232).

Among SCFAs, butyrate is the most efficient suppressor of the allergic immune reaction in the lung. This fatty acid inhibits Th2 and Th9 cell activation (233, 234) and induces T_reg_ cell differentiation (235). It also impedes IgE class switching in B cells (236), mast cell degranulation after FcɛRI activation (237), IL3 and IL-5 secretion by ILC2 cells (238), eosinophilic migration to airways and its chemotaxis, and induces eosinophils apoptosis (239) (Figure 4).

Recent studies using asthmatic murine models have spotlighted the effects of the diet on allergic sensitization and asthma exacerbations. Mice fed with high-fiber diet showed similar effects on allergic inflammation suppression compared to those who were fed with propionate in drinking water (231). Butyrate, but not acetate and propionate, was able to impair infiltration of white cells, particularly eosinophils, and increased FOXP3^+^ cell percentage in the lung, whereas the three SCFAs reduced the reaction after methacholine challenge in an OVA-sensitized mice asthma model (222). High-fiber diet and orally administered acetate suppressed airway inflammation in house dust mite-sensitized mice, inducing tolerance-related M2 macrophage polarization (which produced less IL-4, IL-5 and IL-13 after intranasal challenge) by inhibiting HDAC9, promoting acetylation on Foxp3 promoter (240). Butyrate has also a direct effect on the lung epithelium, reducing the airway remodeling-related collagen deposition on bronchioles in OVA-sensitized mice by impairing expression of matrix metalloproteases 2 and 9, and reduces allergic bronchoconstriction and pulmonary fibrosis by reducing collagen synthesis by lung fibroblasts (241, 242). Butyrate and propionate can repair human lung epithelial damage after protease damage, IL-4, or IL-13 stimulation, but only butyrate reduces IL-6 production by epithelial cells (243).

Even though there is a great number of reports on the effects of gut microbiota-produced and diet SCFAs on the lung epithelium, the effective concentration that reaches the lungs, the inflammation suppression mechanisms, and the effects of novel players in the gut-lung axis are still a matter of study with a therapeutical and preventive perspective, defining gut microbiota as a potential target for new and complementary treatments for asthma and allergic rhinitis.

## Concluding statements

Allergic diseases are exacerbated immunological processes that affect more and more people worldwide, making the development of adequate therapeutic strategies increasingly necessary. Its symptoms are very varied and depend on the characteristics of the individual and include chronic processes such as asthma, atopic dermatitis, rhinitis, and food allergy.

Despite being widely described as an exacerbated Th2 process, several authors coincide on the relevant role played by the epithelial barrier in the allergic process. Epithelial cells form a defensive barrier that, in response to different environmental stimuli, are capable to orchestrate the underlying immune response through secretion of cytokines and chemokines. The epithelial barrier, either from the airways or intestinal compartments, constitutes the first line of cellular defense exposed not only to allergens, but also to various harmful environmental substances. These substances include pollutants and airborne proteases (such as those present in the dust) or, in the case of diet, detergents, emulsifiers and other food additives, conditioning the integrity and correct function of the epithelial barrier. Several *in vitro/in vivo* studies have already related the loss of the integrity of the epithelial barrier to the chronic development of allergic diseases, either due to the action of these agents or due to genetic defects in the junctional proteins keeping the integrity of the barrier. On the contrary, various components of the diet, such as PUFAs, liposoluble vitamins, as well as flavonoids, seem to compensate the deleterious effect of food additives, promoting a correct epithelial function and contributing to reduce the characteristic inflammation of food allergic reactions.

The integrity of the lung and intestinal epithelial barriers is strongly dependent on their respective microbial populations. Alterations in the composition of microbiomes may strongly affect the correct function of these barriers contributing to the appearance of allergic diseases. In this context, we have highlighted the role of a high fiber diet as precursor of intestinal microbiome generation of SCFAs and their importance not only in the intestinal homeostasis but also in anatomically distant environments, with special reference to the gut-lung axis.
